# Comparative microbiome analysis of paired mucosal and fecal samples in Korean colorectal cancer patients

**DOI:** 10.3389/fonc.2025.1578861

**Published:** 2025-06-18

**Authors:** Younggwang Kim, Min Ki Kim, Sanghun Lee

**Affiliations:** ^1^ Department of Bioconvergence & Engineering, Graduate School, Dankook University, Yongin-si, Republic of Korea; ^2^ Department of Surgery, Hallym Hospital, Incheon, Republic of Korea; ^3^ NH Natural Product Institute, Myongji Hospital, Goyang-si, Republic of Korea; ^4^ Department of Biostatistics, Harvard T.H. Chan School of Public Health, Boston, MA, United States

**Keywords:** colorectal cancer, gut microbiota, mucosal microbiota, *Fusobacterium*, *Parvimonas*, *Prevotella 9*, *Holdemanella*, microbial functional pathways

## Abstract

**Background:**

Colorectal cancer (CRC) is increasingly linked to gut microbiome dysbiosis. However, few studies have examined tumor-associated microbial dynamics in Korean CRC patients using both mucosal and fecal samples.

**Methods:**

We analyzed paired fecal and mucosal samples from 30 Korean CRC patients aged 60–80 years before and after surgery. Microbial DNA was sequenced using 16S rRNA gene analysis. Diversity metrics, differential abundance testing (LEfSe), and pathway prediction (PICRUSt2) were performed. Diagnostic performance was evaluated with ROC curves, and associations with clinical parameters were assessed via regression models.

**Results:**

Beta diversity revealed significant compositional differences between fecal and mucosal samples (p = 0.001), with mucosal samples showing higher enrichment of CRC-associated taxa. *Fusobacterium, Prevotella 9, Parvimonas*, and *Holdemanella* were significantly enriched in pre-surgical samples and declined after surgery (p < 0.01). Combined microbial markers yielded an AUC of 0.841 for distinguishing pre- from post-surgical status. Functional predictions indicated upregulation of amino acid metabolism and lipopolysaccharide (LPS) biosynthesis pathways in pre-surgical samples. Notably, *Fusobacterium* abundance correlated with TNM stage (p = 0.028), and *Prevotella 9* abundance decreased with age (p = 0.006).

**Conclusion:**

This study highlights distinct microbial and functional signatures in CRC, particularly from mucosal samples, which offer deeper insights into tumor-microbiota interactions. The identified microbial markers and enriched pathways may contribute to immune modulation and tumor progression. These findings support the potential for microbiome-based diagnostic and therapeutic strategies tailored to Korean CRC patients and underscore the importance of dual-sample analysis in microbiome research.

## Introduction

Colorectal cancer (CRC), the third most common cancer worldwide, presents a significant health challenge with approximately 1.9 million new cases and 935,000 deaths annually (1). Its impact is particularly pronounced in South Korea, where it ranks as the fourth most diagnosed cancer and the third leading cause of cancer deaths (2). Notably, the majority of CRC cases (60–65%) arise in individuals without clear genetic predispositions or family histories, highlighting the critical role of environmental factors and food intake on disease onset (3, 4). Unhealthy dietary choices, such as high intake of red and processed meats and low fiber, along with sedentary lifestyles and obesity, are key contributors to CRC (5). These factors alter the gut microbiota, leading to dysbiosis—an imbalance favoring harmful microbes, which disrupts intestinal homeostasis and promotes inflammation and carcinogenic metabolites, raising CRC risk (5). These findings highlight the role of the gut microbiota in CRC development.

Microbiome alterations have a profound impact on the colorectal cancer (CRC) tumor microenvironment (TME), affecting both tumor progression and responses to therapy. Studies in preclinical models showed that the gut microbiota plays a critical role in both the development and progression of CRC through mechanisms that directly affect epithelial cell transformation and indirectly modulate the immune response (6). Specific microbial species and their virulence factors can activate carcinogenic pathways like Wnt/β-catenin and NF-κB, triggering pro-inflammatory responses and promoting tumor development through DNA damage, altered nutrient availability, and metabolic changes in cancer cells. Within the TME, these microbes influence immune cell function, including T cells and macrophages, and affect cancer-associated fibroblasts and extracellular matrix composition, fostering an immunosuppressive, tumor-promoting environment. Additionally, gut microbiota affects both local and systemic immune responses, altering innate and adaptive immunity in ways that may encourage metastasis, notably through epithelial-mesenchymal transition and gut-vascular barrier disruption (7). Emerging research points to bacteria within the TME as key modulators of CRC metastasis, influencing systemic inflammatory responses and further shaping the tumor’s immune landscape (8). As a result, clinical trials are increasingly examining microbiome-targeted therapies to modulate these responses, potentially enhancing CRC treatments by addressing complications linked to dysbiosis and tumor-promoting bacterial profiles. Recent studies have further identified specific gut microbial subtypes linked to CRC, each with distinct bacterial profiles that correlate with unique clinicopathological features. These subtypes not only enhance our understanding of CRC’s microbial landscape but also highlight promising diagnostic and therapeutic avenues within the microbiome (9). By advancing our understanding of the complex gut-microbiota interactions that facilitate tumor growth and metastasis, these findings open therapeutic opportunities to better target the microbiome in CRC prevention and treatment.

Despite the well-established link between the gut microbiome and CRC, research specifically focused on the gut microbiome in CRC patients within the Korean population remains limited. Recent studies emphasize that factors like geography, ethnicity, and dietary habits profoundly influence gut microbiota composition, leading to distinct microbiome profiles across populations (10, 11). Furthermore, emerging research on aging and gut dysbiosis underscores how age-related microbiome changes interact with inflammatory processes, potentially compounding cancer risks, thus underscoring the importance of demographic-specific studies in understanding CRC pathogenesis within distinct populations (12). Studies involving diverse ethnic groups within the same geographical region indicate that local dietary habits and cultural practices can lead to distinct microbiome profiles, highlighting the need for population-specific research. Such targeted research would address unique microbial and environmental interactions relevant to the Korean context, contributing to more effective, culturally informed approaches to CRC management.

We hypothesize that by analyzing both mucosal and fecal samples, we can identify distinct microbial profiles associated with CRC progression, which may differ based on their proximity to the TME. This approach may reveal unique microbial markers relevant to CRC diagnosis and therapy. While fecal samples broadly represent the luminal microbiota, mucosal samples capture microbial communities directly interacting with the tumor environment (13). Research suggests that fecal samples may miss certain microbial species that play critical roles in CRC progression, whereas mucosal samples—by their proximity to tumor sites—offer unique insights into the specific microbial populations involved in cancer-related processes. This approach allows us to investigate how the microbiota within the TME may contribute to cancer growth and immune modulation, highlighting the added value of analyzing tissue-associated microbiota alongside luminal samples. This dual-sampling strategy, therefore, enhances our understanding of the microbiome’s involvement in CRC and supports the development of microbiome-based diagnostic and therapeutic strategies.

## Materials and methods

### Volunteer recruitment and inclusion/exclusion criteria

We enrolled volunteers diagnosed with primary CRC at our institution between July 1, 2021, and March 2, 2023. Participants were selected based on specific inclusion and exclusion criteria to ensure the homogeneity and relevance of the study cohort. Eligible participants were between 60 and 80 years of age and presented for treatment upon the diagnosis of primary CRC. The exclusion criteria were as follows: individuals who used steroids or immunosuppressants within six months prior to surgery; severe communication difficulties such as dementia or intellectual disability; American Society of Anesthesiologists (ASA) score of III or higher; Eastern Cooperative Oncology Group (ECOG) performance status of > 3; and current pregnancy. Additionally, we excluded individuals with underlying conditions that could affect their immune status, including rheumatoid arthritis, Behçet’s disease, or inflammatory bowel disease. This rigorous selection process was designed to minimize the variability and enhance the reliability of our findings. Additionally, all the participants provided informed consent, ensuring ethical compliance and patient understanding of the aims and procedures of the study. Ethics approval was granted by the Institutional Review Board (IRB 2021-05–005 and 2020-01-016) of Myongji Hospital.

### Sample collection and DNA extraction

Participants provided fecal samples before PEG bowel preparation, preserved in DNA/RNA protector reagent (New England Biolabs), transported on an icepack to the laboratory within 12 h, and stored at –80°C before use. During surgery, mucosal tissues were collected and stored at –80°C until use. Genomic DNA from fecal samples was extracted using the QIAamp PowerFecal Pro DNA Kit (Qiagen), and DNA from mucosal tissues was extracted using the QIAamp Fast DNA Tissue Kit (Qiagen) according to the manufacturer’s instructions with slight modifications, which included heating samples at 95°C for 10 minutes to ensure thorough cell disruption, particularly for Gram-positive bacteria, and subjecting the samples to bead-beating for 10 minutes using a vortex adapter to enhance microbial DNA yield. DNA purity was assessed using a NanoDrop 2000 spectrophotometer (Thermo Fisher Scientific). DNA integrity was confirmed by electrophoresis on a 2% (w/v) agarose gel at 180 V for 30 min in Tris-borate-EDTA buffer. We stored all DNA samples at –20°C until they were required for subsequent analysis.

### Bioinformatics and statistical analysis

Raw sequencing data were processed using QIIME2 (version 2023.9). Sequences were demultiplexed based on unique barcode sequences, and quality filtering was performed using the DADA2 plugin in QIIME2 to denoise sequences, remove low-quality reads (Phred score < 20), and truncate reads where quality scores dropped significantly. Primer sequences were trimmed, and reads were truncated at positions where the median quality score fell below 25. Chimeric sequences were identified and removed using the consensus method in DADA2. Amplicon sequence variants (ASVs) were generated using DADA2, providing high-resolution identification of unique sequences differing by as little as one nucleotide. Taxonomy was assigned to ASVs using a pre-trained naïve Bayes classifier against the SILVA 138.1 reference database, specific to the V3–V4 region of the 16S rRNA gene. Alpha diversity, representing within-sample diversity, was assessed using the Observed ASVs and Shannon diversity index, and non-parametric tests (e.g., Kruskal-Wallis) were applied due to the non-normal distribution of the data. Beta diversity, representing between-sample diversity, was calculated using unweighted UniFrac distances, and principal coordinates analysis (PCoA) plots were generated to visualize clustering patterns. Permutational Multivariate Analysis of Variance (PERMANOVA) with 999 permutations was used to test for significant differences between groups. Differential abundance of taxa between groups was analyzed using Linear Discriminant Analysis Effect Size (LEfSe), with a logarithmic LDA score threshold of ≥ 3.0 and *p* ≤ 0.05. Further statistical analyses were performed using R (version 4.0.2) and Python (version 3.8). Receiver operating characteristic (ROC) curves were generated using the Random Forest algorithm to assess the diagnostic potential of microbial markers. Associations between microbial taxa and clinical parameters were examined through stepwise regression analysis, guided by the Akaike Information Criterion (AIC), and p-values were adjusted for multiple comparisons using the False Discovery Rate (FDR) method. Statistical significance was set at *p* ≤ 0.05 for all analyses. Functional pathway prediction was performed using PICRUSt2 (version 2.5.1) to infer metagenomic functions based on the 16S rRNA gene data. Pathway abundance was visualized using STAMP (version 2.1.3) to identify significant differences in metabolic and biosynthetic pathways between sample groups. The Bonferroni method was applied to adjust *p*-values, with a significance threshold set at an adjusted *p*-value of 0.05. Pathways were ranked by effect size to identify the most enriched functions.

## Results

### Profile and clinical characteristics of the Korean CRC cohort in this study

Our study evaluated a cohort of 30 Korean patients diagnosed with CRC, with a mean age of 68.8 years (± 8.7) and a mean body mass index of 24.0 (± 3.0). Our study cohort consisted of 36.7% females who presented with comorbidities, including diabetes (46.7%), hypertension (56.7%), and hyperlipidemia (43.3%). The cancer stages of the patients at diagnosis were predominantly stages II (36.7%) or III (46.7%). Carcinoembryonic antigen (CEA) levels decreased significantly from a preoperative mean of 22.8 ng/mL (± 72.2) to a postoperative mean of 3.6 ng/mL (± 2.7). The clinical details are presented in **Table 1**.

**Table 1:** Overview of the demographic and clinical characteristics of a cohort of pre-surgical Korean patients diagnosed with colorectal cancer.

Clinical parameter	Colorectal Cancer
Age (Mean ± SD)	68.8 ± 8.7
BMI (Mean ± SD)	24.0 ± 3.0
Gender (n, (%))	
Female	11 (36.7)
Comorbidity	
Diabetes mellitus (n (%))	14 (46.7)
Hypertension (n (%))	17 (56.7)
Hyperlipidemia (n (%))	13 (43.3)
Smoke (n, (%))	13 (43.3)
Alcohol (n, (%))	10 (33.3)
Tumor location (n, (%))	
Right side	15 (50.0)
Left side	15 (50.0)
TNM stage (n, (%))	
I	4 (13.3)
II	11 (36.7)
III	14 (46.7)
IV	1 (3.3)
Metastatic Lymph node (n, (%))	
negative	14 (46.7)
positive	16 (53.3)
Perineural invasion (n, (%))	9 (30.0)
K-RAS (n, (%))	12 (40.0)
CEA (Mean ± SD)	22.8 ± 72.2
Adjuvant chemotherapy (n, (%))	16 (53.3)
Probiotic intake (n, (%))	18 (60.0)

Data are presented as the mean ± SD for continuous variables and counts (percentages) for categorical variables.

For the microbial composition analysis, we collected paired mucosal tissue samples from tumor-adjacent sites (T1), as well as fecal samples pre-surgery (S1) and at 6–12 months post-surgery (S2).

### Microbial richness and diversity dynamics

**Figure 1:**
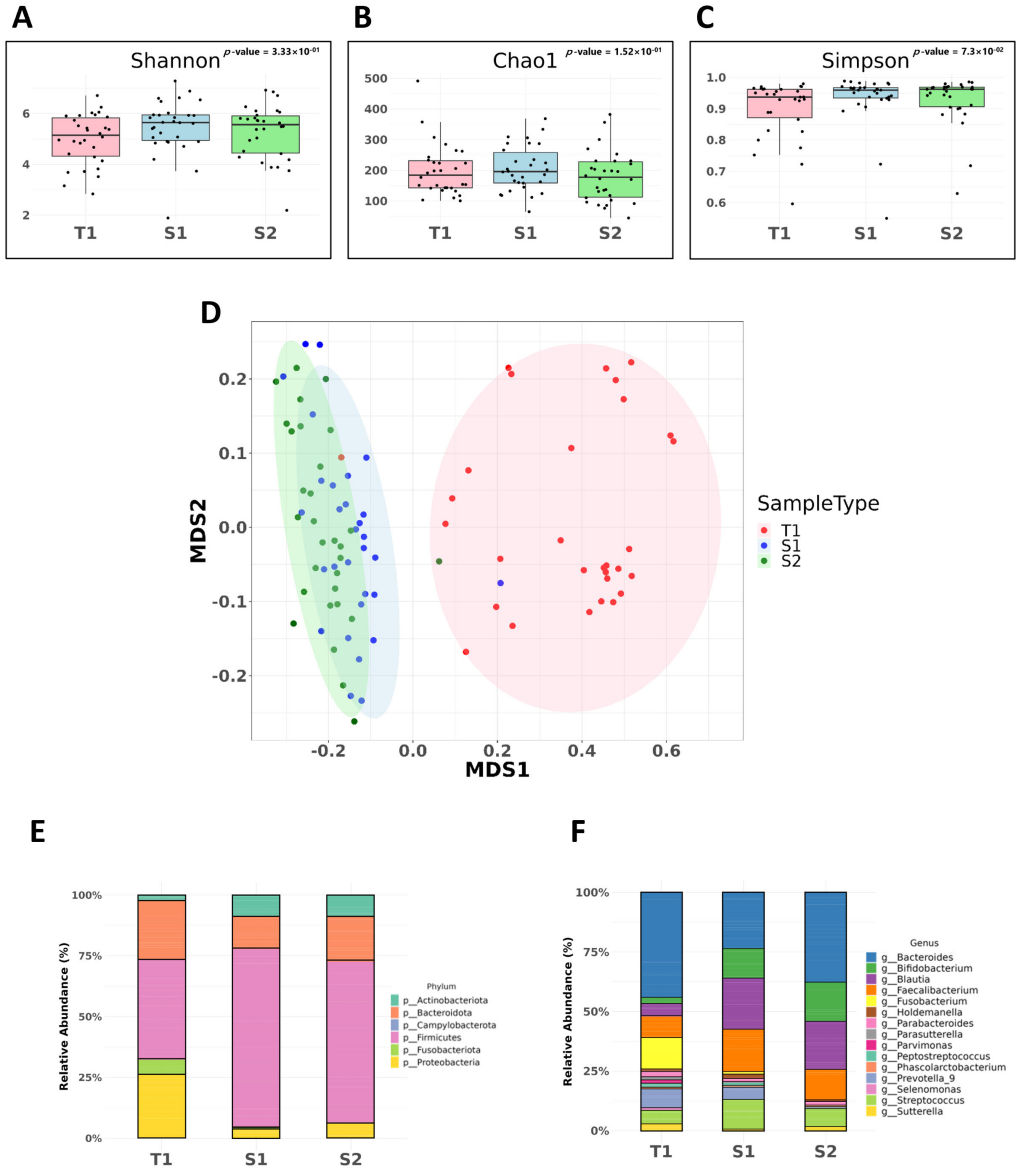
Microbial diversity and comparative analysis of bacterial microbiota in CRC samples. These samples include pre-surgical fecal samples (S1), mucosal tissue samples (T1), and post-surgical fecal samples (S2). **(A)** Shannon Index. Assessment of alpha-diversity using the Shannon index across fecal and mucosal CRC samples. **(B)** Chao1 Index. Chao1 index values indicating species richness within fecal and mucosal CRC samples. **(C)** Simpson Index. Simpson index values used to evaluate the probability of encountering identical microbial species within CRC samples. **(D)** Non-metric Multidimensional Scaling (NMDS) with Unweighted UniFrac. Beta-diversity analysis using NMDS and the unweighted UniFrac method, showing significant differences in microbial community composition between fecal and mucosal samples (*p* = 0.001). **(E)** Comparative analysis at the phylum level. **(F)** Comparative analysis at the genus level.

The microbial richness and diversity within our CRC patient cohort were assessed using alpha- diversity indices (Shannon, Chao1, and Simpson indices). These indices aimed to identify significant differences in microbial diversity between fecal and mucosal samples. Our analysis showed no significant variation in microbial diversity across different sample types, as indicated by the Shannon (*p* = 0.152), Chao1 (*p* = 0.333), and Simpson indices (*p* = 0.073) (**Figures 1A–C**, respectively), suggesting a consistent level of microbial diversity.

However, beta diversity analysis, which evaluates variations in the microbial community composition between samples, revealed significant differences. Using Non-metric Multidimensional Scaling (NMDS) along with the unweighted UniFrac method, we observed a marked difference in the microbial community composition between fecal and mucosal samples (*p* = 0.001), underscoring the unique microbial profiles in mucosal samples compared to fecal samples (**Figure 1D**).

### Comparative analysis of the bacterial microbiota across different CRC sample types

Our comparative analysis at the phylum level revealed significant variations in bacterial composition across different sample types. S2 samples were predominantly composed of *Firmicutes* (65.9%), followed by *Bacteroidota* (8.3%) and *Proteobacteria* (5.91%). In contrast, S1 samples had a higher proportion of *Firmicutes* (73.3%) and fewer *Bacteroidota* (13.4%) and *Proteobacteria* (3.95%) than S2 samples. T1 samples presented a distinct microbial landscape, with a higher presence of *Bacteroidota* (24.5%), *Proteobacteria* (26.7%), and a notable proportion of *Fusobacteriota* (6.13%) (**Figure 1E**).

Significant shifts were also observed at the genus level. *Fusobacterium* was enriched in T1 samples (14.57%), markedly higher than in S1 (0.79%) and S2 (0.04%) samples. Additionally, *Prevotella 9* was more prevalent in T1 (9.05%) and S1 (6.13%) samples, but was less abundant in S2 samples (0.78%). *Blautia* was less abundant in T1 samples (6.3%) than in S1 (23.9%) and S2 (22.3%) samples (**Figure 1F**).

### Identifying key microbial markers in CRC following colorectal surgery

**Figure 2:**
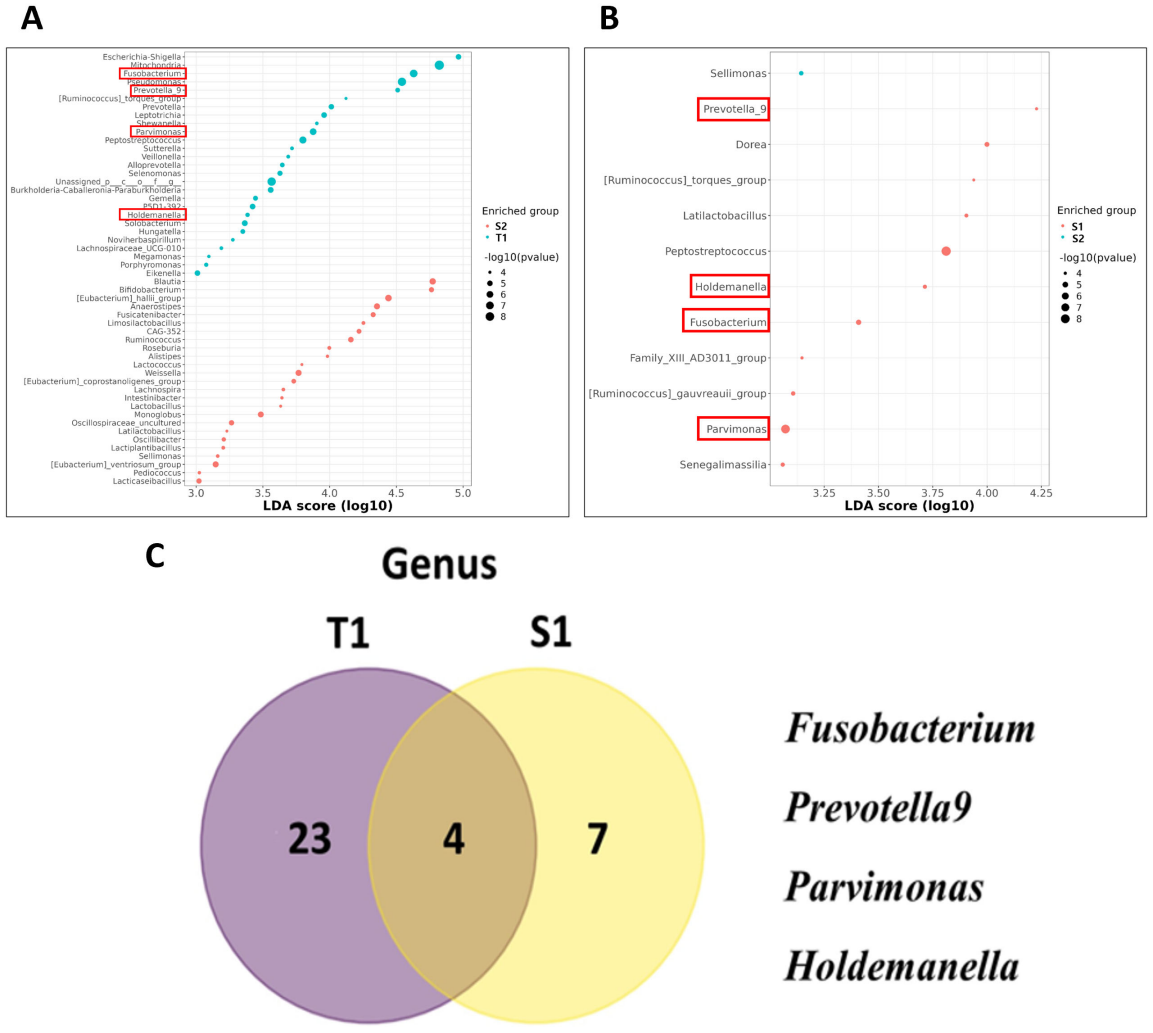
Utilizing linear discriminant analysis effect size (LEfSe) to explore microbial taxa differences between pre-surgery and post-surgery CRC patient samples. **(A)** Genus Level Enrichment in tissue samples (T1) Samples. **(B)** Genus Level Enrichment in S1 Samples. **(C)** Venn diagram represented the number of markers for CRC at the Genus Level between T1 and S1 Samples.

To identify potential biomarkers prevalent in CRC patient samples, we employed Linear discriminant analysis Effect Size (LEfSe) to compare the microbial taxa between the pre- and post-surgery groups. In T1 samples, we observed a notable enrichment of *Proteobacteria* and *Fusobacteriota* at the phylum level (**Supplementary Figure 1A**). At the genus level, various microorganisms, including *Escherichia-Shigella*, *Fusobacterium*, *Pseudomonas, Prevotella 9*, *Parvimonas*, *Peptostreptococcus*, *Sutterella*, *Selemonas*, and *Holdemanella* demonstrated dominance in T1 samples (**Figure 2A**). Conversely, in the S1 samples, the phyla *Firmicutes* and *Fusobacteriota* were significantly enriched (**Supplementary Figure 1B**), with a genus-level dominance of *Prevotella 9*, *Dorea*, *Peptostreptococcus*, *Holdemanella*, *Fusobacterium*, and *Parvimonas* compared to the S2 samples (**Figure 2B**). Venn diagram analysis focusing on the cross-examination between S1 and T1 samples revealed *Fusobacteriota* as a common enriched taxon at the phylum level in both sample types, indicating its significant presence in CRC-associated microbial communities (**Supplementary Figure 1C**). At the genus level, *Fusobacterium*, *Parvimonas*, *Prevotella 9*, and *Holdemanella* were the most abundant in both sample types (**Figure 2C**).

### Temporal dynamics of bacterial taxa across paired samples in patients with CRC

**Figure 3:**
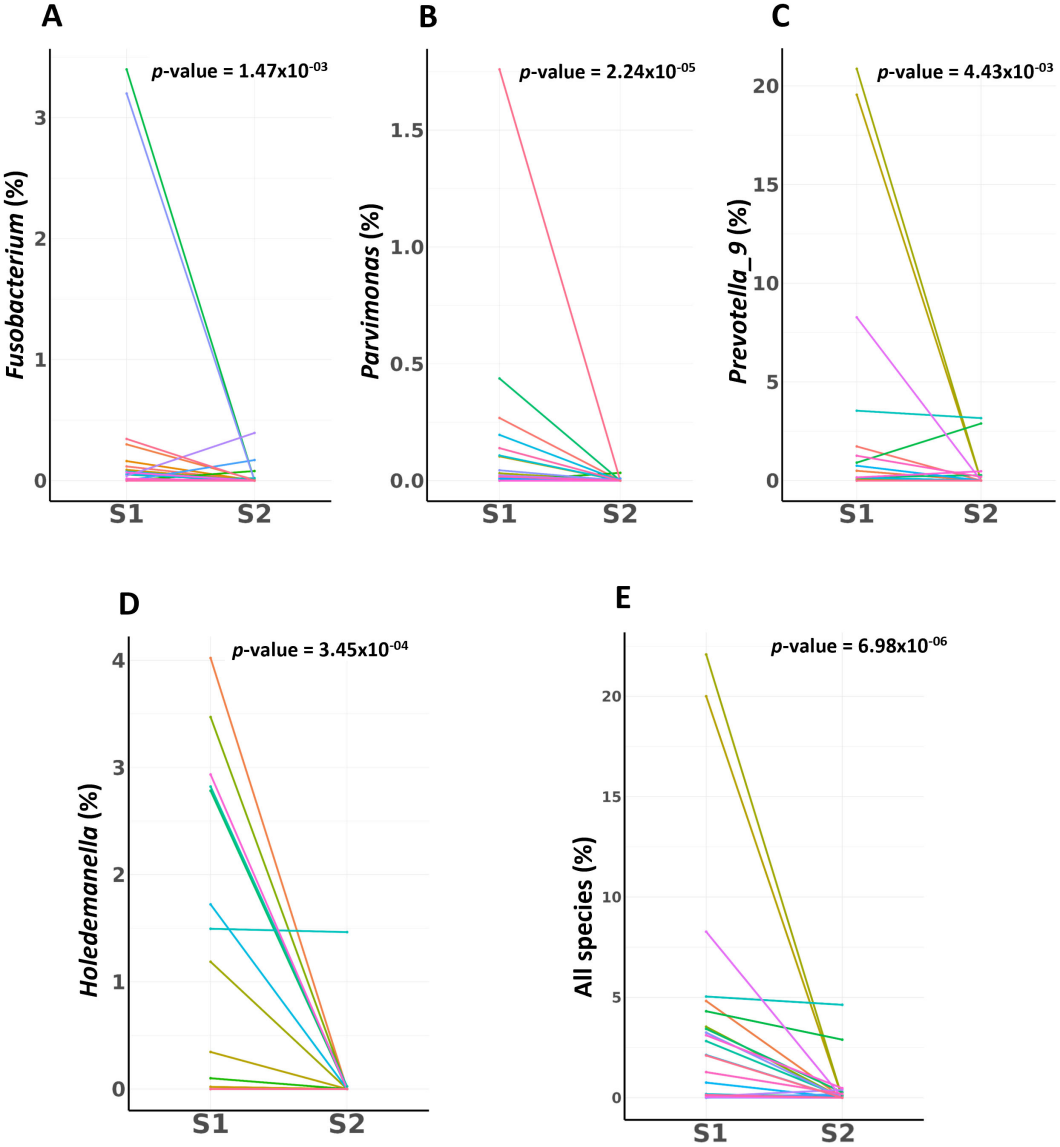
Employing the Wilcoxon rank-sum test, this analysis quantifies the significant shifts in bacterial abundance observed between pre-surgical (S1) and post-surgical (S2) samples in CRC patients. **(A)** *Fusobacterium* Abundance Shift **(B)** *Parvimonas* Abundance Shift **(C)** *Prevotella 9* Abundance Shift **(D)** *Holdemanella* Abundance Shift **(E)** Combined Microbial Dynamics. Key biomarkers for CRC combination shows a profound and statistically powerful decrease from pre-surgery to post-surgery.

Our investigation of the temporal dynamics of specific bacterial taxa across S1 and S2 samples in patients with CRC revealed significant shifts in bacterial abundance between these two phases using the Wilcoxon rank-sum test. The taxa *Fusobacterium* (*p* = 1.47×10^-3^), *Parvimonas* (*p* = 2.24×10^-5^), *Prevotella 9* (*p* = 4.43×10^-3^), and *Holdemanella* (*p* = 3.45×10^-4^), all demonstrated substantial decreases in abundance from the S1 to the S2 phase. The combination of four key microbes—*Prevotella 9*, *Holdemanella*, *Parvimonas*, and *Fusobacterium*—exhibited a significant decrease in abundance post-surgery in patients with CRC (*p* = 6.98×10^-06^) (**Figures 3A–E**).

### Diagnostic potential of the identified genera

**Figure 4:**
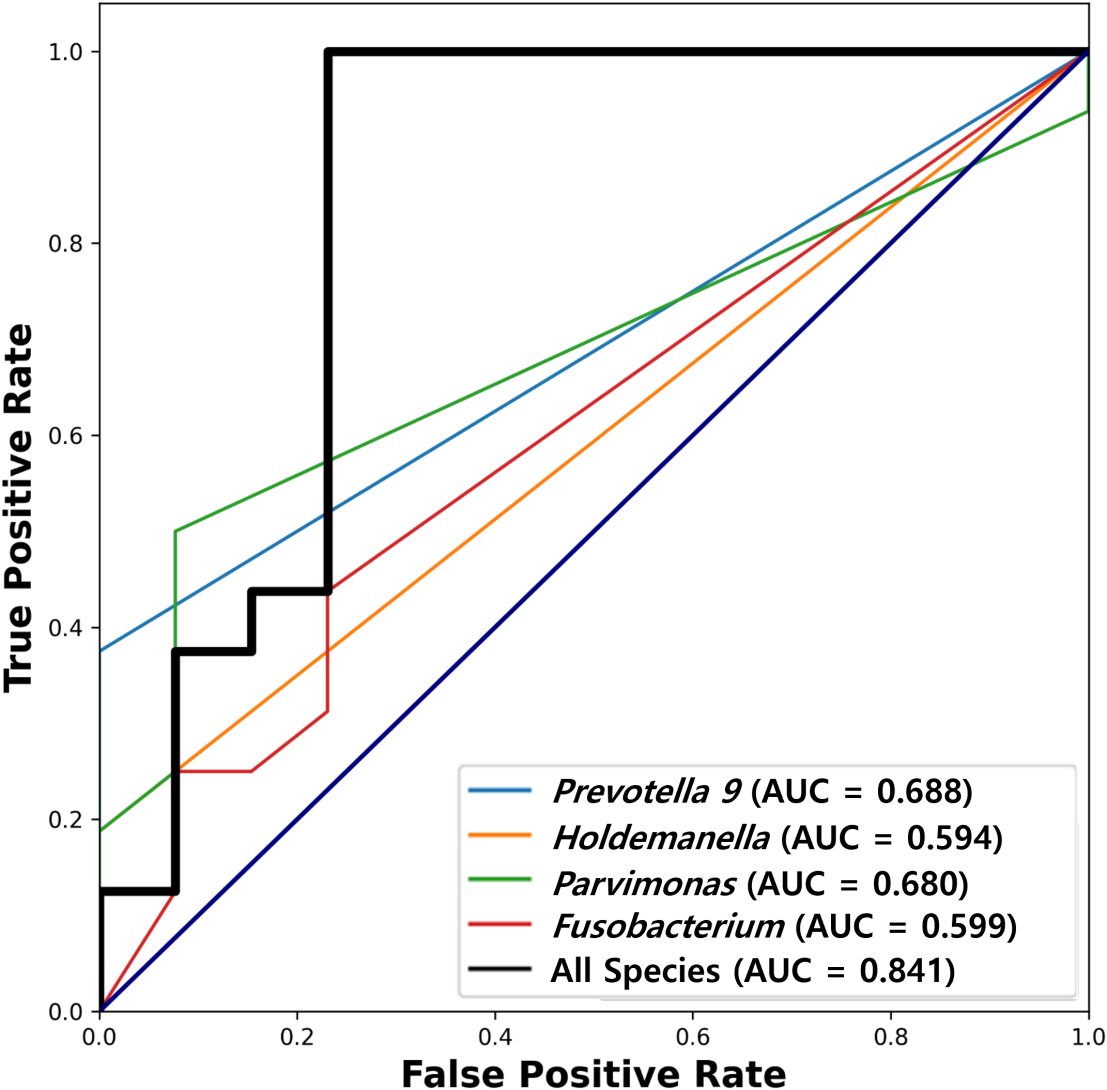
Receiver operating characteristic (ROC) analysis of diagnostic potential. ROC analysis utilized to evaluate the discriminative ability of *Prevotella 9*, *Holdemanella*, *Parvimonas*, and *Fusobacterium* between pre-surgical (S1) and post-surgical (S2) CRC patient samples. Individual Area Under the Curve (AUC) values are 0.688 for *Prevotella 9*, 0.594 for *Holdemanella*, 0.680 for *Parvimonas*, and 0.599 for *Fusobacterium*, indicating varying levels of diagnostic potential. The combined analysis of these species shows a higher AUC (0.841), highlighting their robust potential as microbial markers for distinguishing between different period of CRC patient.

The diagnostic potential of these genera, as assessed using ROC curve analysis, varied. *Prevotella 9*, *Holdemanella*, *Parvimonas*, and *Fusobacterium* showed individual AUC values of 0.688, 0.594, 0.680, and 0.599, respectively, suggesting varying degrees of discriminative ability between S1 and S2 samples. A combined analysis of these species achieved a higher AUC, indicating the robust potential of these microbial markers to distinguish between CRC stages (**Figure 4**).

### Associations between microbial abundance and clinical parameters

By exploring the connections between clinical parameters and microbial abundance, our study utilized stepwise regression analysis, fine-tuned by the Akaike Information Criterion (AIC), and adjusted for multiple comparisons using the False Discovery Rate (FDR) method. We identified a significant inverse relationship between age and the abundance of *Prevotella 9* in T1 samples, suggesting an age-associated decrease in the abundance of this microorganism (β = -0.653, 95% CI [confidence interval];: -1.025 to -0.281, *p* = 6.1×10^-3^) (**Supplementary Table 1**). High lipid levels were significantly correlated with the abundance of *Holdemanella* in T1 samples (β = -5.747, 95% CI: -10.923 to -0.571, *p* = 2.72×10^-2^) (**Supplementary Table 4**). Additionally, a marginal association was noted between CEA levels and the abundance of *Prevotella 9* in T1 samples (β = 7.034, 95% CI: -0.736 to 14.804, p = 6.39×10^-2^) (**Supplementary Table 1**). TNM stage was found to influence the abundance of *Fusobacterium* in S1 samples (β = 0.610, 95% CI: 0.066 to 1.154, *p* = 2.80×10^-2^) (**Supplementary Table 2**), while CEA levels had a nominally significant association with the abundance of *Holdemanella* in S1 samples (β = -3.977, 95% CI: -8.161 to 0.20], *p* = 5.76×10^-2^) (**Supplementary Table 4**). Our data did not reveal any significant correlations between the abundance of *Parvimonas* and clinical parameters (**Supplementary Table 3**).

### Functional pathway analysis between pre-surgical and post-surgical samples in CRC patients

**Figure 5:**
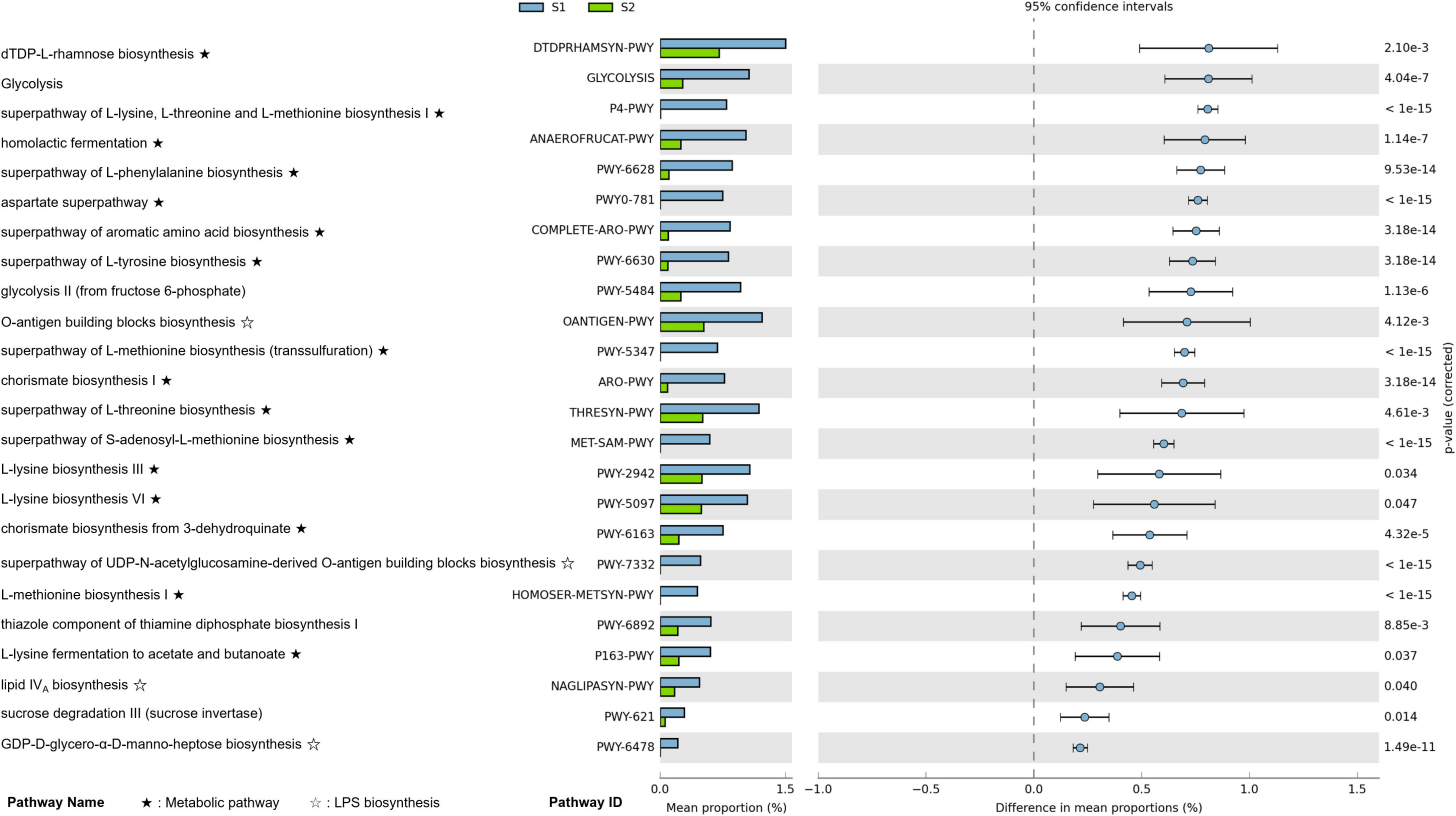
Differential functional pathway enrichment between pre-surgical (S1) and post-surgery (S2) fecal samples in CRC patients (*p*-value corrected < 0.05). Functional pathway enrichment analysis shows the enriched metabolic and biosynthetic pathways in S1 compared to S2 fecal samples, based on PICRUSt2 analysis. Pathways are ranked by effect size, with key pathways such as amino acid biosynthesis, and lipopolysaccharide (LPS) biosynthesis significantly enriched in S1. Error bars represent the 95% confidence intervals for differences in pathway proportions.

We conducted a functional pathway enrichment analysis using PICRUSt2 predictions and MetaCyc pathway annotations to investigate the differences between pre-surgical (S1, T1) and post-surgical (S2) sample types in CRC patients (**Figure 5**) (**Supplementary Figure 2**). Adjusted p-values were calculated using the Bonferroni method with a cutoff of 0.05, and pathways were arranged by effect size.

In pre-surgical samples, the most enriched pathways included *dTDP-L-rhamnose biosynthesis*, *glycolysis*, *the superpathway of L-lysine*, *L-threonine, and L-methionine biosynthesis I*, and *homolactic fermentation*. Across these pre-surgical samples, we observed a significant enrichment in amino acid metabolism pathways, particularly those related to L-lysine, phenylalanine, L-threonine, L-methionine, tyrosine, aspartate, and lactic acid metabolism. Additionally, pathways associated with lipopolysaccharide (LPS) biosynthesis were enriched in pre-surgical samples. Key pathways in this category included the *O-antigen building blocks biosynthesis*, *the superpathway of UDP-N-acetylglucosamine-derived O-antigen building blocks biosynthesis*, *lipid IV_A_ biosynthesis*, *GDP-D-glycero-α-D-manno-heptose biosynthesis*, and *CMP-3-deoxy-D-manno-octulosonate biosynthesis*.

## Discussion

Our analysis of Korean CRC patients reveals significant differences in the microbial composition between fecal and mucosal samples, suggesting potential roles of gut microbiota in CRC pathogenesis. In our cohort, candidate markers, including *Fusobacteriota* and the genera *Fusobacterium*, *Prevotella 9*, *Parvimonas*, and *Holdemanella*, were identified. The consistent presence of these microbes across the different sample types suggests they may play roles in CRC development. In our analysis, we observed that well-known CRC-associated species, such as *Fusobacterium* and *Parvimonas*, exhibited high abundance in both T1 and S1 samples, with a notably higher concentration in T1 samples. It suggests that S1 samples may serve as a surrogate for T1 samples, indicating a consistent microbial profile potentially implicated in CRC pathogenesis. The elevated presence of these microbes specifically in T1 samples underscores their potential role within the TME and highlights the importance of analyzing both sample types to capture comprehensive microbial dynamics.

Previous studies conducted have shown that mucosal samples, due to their proximity to the tumor site, reveal microbial species more closely associated with the tumor environment (14). For instance, genera such as *Finegoldia* and *Porphyromonas* have been found more abundantly in mucosal tissues compared to luminal samples, highlighting the value of tissue-based sampling for capturing species directly interacting with the tumor. Additionally, a study involving CRC patients identified *Eubacterium ramosum* (ER) as a potential biomarker (15). Using quantitative PCR on mucosal and fecal samples, followed by ROC curve analysis, this study demonstrated a significant enrichment of *ER* in mucosal samples (control vs. case; AUC = 0.789, *p* = 0.002) compared to fecal samples (control vs. case; AUC = 0.650, *p* = 0.113) among CRC patients. These findings reinforce the idea that mucosal samples offer a more precise reflection of the microbiota’s involvement in CRC. Therefore, incorporating mucosal samples alongside fecal samples in our analysis substantially enhances the depth of our investigation into the microbial communities that interact directly with the gut epithelium.

Our findings align with those of a similar study, which demonstrated that *Fusobacterium*, *Parvimonas*, and *Prevotella* (*Prevotella 9* in our case) are more enriched in intratumoral tissues compared to adenomas, with associations noted between *KRAS* mutations (*p* < 0.001) and microsatellite instability (*p* < 0.001) in these samples (16). In *KRAS*-mutant CRC cells, recent research has highlighted distinct metabolic adaptations that support tumor growth (17). Specifically, these cells develop a heightened reliance on amino acids such as glutamine and leucine to sustain energy metabolism, redox balance, and macromolecule synthesis. *KRAS* mutations increase the expression of amino acid transporters (AATs) enhancing amino acid uptake. Furthermore, Yes-associated protein 1 has been shown to promote AAT expression and CRC progression through mTOR pathway in *KRAS*-mutant cells. In line with these findings, our functional analysis revealed an upregulation of several amino acid metabolism pathways in both S1 and T1 samples, suggesting elevated amino acid turnover within the TME (**Figure 5**). Building on this, our functional analysis revealed that specific metabolic pathways associated with metabolite production were upregulated in pre-surgical samples, pointing to an enhanced turnover of metabolites essential to CRC progression. Pathways involving the metabolism of amino acids such as L-lysine, phenylalanine, L-threonine, L-methionine, tyrosine, aspartate, and lactic acid were notably enriched, which aligns with the increased amino acid dependency observed in pre-surgical samples. These metabolites not only support biosynthetic needs for cell proliferation but also facilitate immune evasion within the TME, creating favorable conditions for CRC pathogenesis (18).

In addition, our functional pathway analysis also identified a significant enrichment of LPS biosynthesis pathways in pre-surgical samples, underscoring an additional microbial-driven mechanism that may contribute to CRC progression. By applying a stringent Bonferroni correction for multiple comparisons, we strengthened the statistical robustness of these findings, ensuring confidence in the role of LPS biosynthesis pathways in the TME. The LPS biosynthesis pathways identified include the *O-antigen building blocks biosynthesis*, *lipid IV_A_ biosynthesis*, *GDP-D-glycero-α-D-manno-heptose biosynthesis*, and *CMP-3-deoxy-D-manno-octulosonate biosynthesis* (**Figure 5**). These pathways play a central role in creating a pro-inflammatory environment within the gut by activating the TLR4-NF-κB signaling cascade, which in turn induces the secretion of inflammatory cytokines. This inflammatory response not only disrupts the gut barrier but also facilitates immune evasion, potentially promoting tumor initiation and progression within CRC. The prominence of LPS biosynthesis specifically in T1 samples suggests that mucosal-associated microbes are particularly influential in fostering inflammation around the tumor site. In this context, LPS may enhance TLR4 receptor expression and drive chronic inflammation, further supporting tumor growth and potentially contributing to metastasis. This observation confirms the importance of including mucosal samples in analyses, as they may capture microbial activities that are more directly involved in tumor-specific interactions and inflammation.

Our study demonstrated that *Fusobacterium* was significantly more abundant in pre-surgical samples (S1, T1), aligning with previous findings that link its presence to CRC progression (**Figure 1F**). Previous research suggests that *Fusobacterium* colonization in tumor tissue may induce DNA alterations in genes like *ATM* and *PIK3CA*, which play roles in regulating the cell cycle and influence downstream proteins, including tumor suppressors such as p53 and BRCA1 (19). Further studies highlight that *Fusobacterium nucleatum* contributes to CRC progression by driving processes like epithelial-mesenchymal transition (EMT), modifying the TME, and influencing oncogenic noncoding RNAs, which together enhance CRC cell migration and invasion. In our study, regression analysis indicated a significant association between *Fusobacterium* abundance and TNM staging, with higher levels of *Fusobacterium* positively influencing cancer stage (β = 0.610, 95% CI: 0.066 to 1.154, *p* = 2.80×10^-2^) (**Supplementary Table 4**). Other studies have indicated that differences in the abundance of *Fusobacterium nucleatum* correlate with staging, suggesting that its metastatic characteristics may impact this association (20–22).

*Parvimonas*, like *Fusobacterium*, was significantly enriched in the pre-surgery group and is known to promote CRC development as an anaerobe. Interestingly, one study has correlated the colonization of *Parvimonas micra* with decreased survival rates in patients with CRC (23). Additionally, *in vivo* tests have reported that *Parvimonas* enhances tumorigenesis through the epigenetic reprogramming of human intestinal cells and improves the Th17-mediated immune response in the colon (24).

*Prevotella*, encompassing over 50 distinct species, has traditionally been regarded as commensal due to its prevalence in the healthy human gut, particularly among individuals consuming a fiber-rich diet (25). However, recent studies suggest that certain strains of *Prevotella* may exhibit pathobiontic behavior, contributing to immune dysregulation and systemic inflammatory diseases, including periodontitis, bacterial vaginosis, rheumatoid arthritis, and metabolic disorders (26). For example, *Prevotella copri* has been implicated in autoimmune conditions such as rheumatoid arthritis and shown to exacerbate inflammation in colitis models (27, 28). Moreover, the genus has been proposed as a potential *In vivo* diagnostic marker for CRC (29, 30).

Similarly, *Holdemanella*, typically considered to have anticancer properties (31), was unexpectedly abundant in CRC patients in our study (**Figure 1F**). Although these genera have been mentioned in prior CRC-related microbiome studies, their co-enrichment in both mucosal and fecal samples within our Korean cohort is not well documented. This observation may reflect population-specific microbial patterns or context-dependent shifts related to disease state. However, given the limited functional characterization of *Prevotella 9* and *Holdemanella* in CRC, their roles remain unclear. Further studies are required to determine whether their presence reflects causal involvement in tumor biology or a response to changes in the tumor microenvironment. Increasing attention has been paid to the dual roles of commensal microbes, which may act as opportunistic pathogens under certain conditions (32, 33). In the context of CRC, immune modulation, metabolic reprogramming, and altered ecological niches within the tumor microenvironment may favor such transitions, potentially facilitating disease progression. Understanding how these taxa adapt to or exploit the TME will be essential in clarifying their relevance to CRC pathogenesis.

Our study suggests microbial markers with potential to enhance CRC screening and diagnosis, particularly through non-invasive methods. While the AUC values of individual genera (ranging from 0.594 to 0.688) suggest modest discriminative capacity, the combined model demonstrated a stronger signal (AUC = 0.841). However, caution is warranted in interpreting these results as clinically actionable biomarkers until validated in independent cohorts. The consistent detection of specific microbial markers in Korean CRC patients, including findings that align with existing studies on the high abundance of *Fusobacterium nucleatum*, reinforces their role as reliable indicators for disease progression and prognosis (34). Prior Korean studies have similarly shown that elevated *Fusobacterium nucleatum* abundance is associated with poor survival outcomes, substantiating our observations on the prognostic value of this specific microbial marker (35). Notably, the fecal microbial signatures identified in this study could serve as biomarkers for early-stage disease detection, reflecting microbial dysbiosis typically associated with CRC (36, 37). Liang et al. demonstrated that the non-invasive diagnosis of CRC using fecal bacterial markers, including *Fusobacterium nucleatum* and *Bacteroides clarus*, significantly improved diagnostic accuracy when used alongside fecal immunochemical tests (38).

Despite the significant findings, our study has several limitations that should be taken into account when interpreting the results. First, the relatively small sample size of 30 elderly Koreans, although adequate for identifying significant associations within them, limits the generalizability of our findings. Typically, larger sample sizes are necessary to detect associations with smaller effect sizes, and this study may lack the statistical power required to identify such associations, particularly in subgroup analyses. Furthermore, expanding this research to include multi-ethnic and geographically diverse cohorts would enhance both the statistical power and the external validity of our findings. Second, with 63.3% male and 36.7% female participants, our study may not fully represent the microbial profiles across the entire Korean population of patients with CRC (39). However, our cohort consists primarily of elderly patients who have been exposed to longstanding Korean lifestyle and environmental factors, potentially reflecting a distinct Korean gut microbiome profile. Third, our study included a patient group labeled S2, who were sampled between 6 and 12 months postoperatively. Although this timeframe provides meaningful insight into the intermediate effects of surgical and therapeutic interventions, longer follow-up periods would be beneficial for capturing the sustained and long-term impact on microbiota composition. Additionally, the potential effects of chemotherapy during this interval cannot be ruled out (40); however, our paired-sample design was employed specifically to mitigate the influence of such confounders. Forth, although we observed correlations between microbial abundance and CRC, no experimental validation was conducted to establish causative relationships, which limits the interpretation of these findings. Further mechanistic studies are warranted to confirm the functional roles of the identified microbes in CRC progression. Fifth, while our study centers on profiling the microbiome in relation to clinical parameters, data on broader lifestyle factors such as dietary habits, medication use, and physical activity were not included. Future studies that incorporate these variables could offer more contextual insight into the observed microbiome patterns. Finally, our reliance on 16S rRNA gene sequencing predominantly allowed identification at the genus level and occasionally at the species level. This limitation hinders the precise identification of the specific bacterial strains involved in CRC. Therefore, we plan to perform follow-up studies employing shotgun metagenomic sequencing, which provides higher-resolution data and enables more detailed investigations into the functional and taxonomic characteristics of CRC-associated microbial communities.

Our study identifies specific microbial candidate markers and metabolic pathways associated with CRC progression, underscoring their potential roles in diagnosis, prognosis, and treatment strategies. The consistent presence of taxa such as *Fusobacterium*, *Prevotella 9*, *Parvimonas*, and *Holdemanella* suggests they may contribute to CRC development through immune modulation, metabolic reprogramming, and interactions within the TME. Our functional analysis further reveals enriched metabolic pathways, highlighting microbial-driven processes that may support immune evasion and facilitate a tumor-promoting environment, particularly through amino acids and LPS biosynthesis pathways. These findings provide a foundation for future research on microbiome-targeted strategies in CRC management and suggest the potential for developing non-invasive diagnostic tools based on fecal microbial signatures. Future studies should validate these findings in larger cohorts across different ethnic and geographic populations to confirm the diagnostic utility and population specificity of these microbial markers.

## Data Availability

The original contributions presented in the study are included in the article/**Supplementary Material**. Further inquiries can be directed to the corresponding author.

## Supplementary material

Supplementary Figure 1 Utilizing Linear Discriminant Analysis Effect Size (LEfSe) to Explore Microbial Taxa Differences Between Pre-Surgery and Post-Surgery CRC Patient Samples. **(A)** Phylum Level Enrichment in tissue samples (T1) Samples. **(B)** Phylum Level Enrichment in S1 Samples. **(C)** Venn diagram represented the number of markers for CRC at the Phylum Level between T1 and S1 Samples.

Supplementary Figure 2 Differential Functional Pathway Enrichment Between Mucosal (T1) and Post-Surgery Fecal (S2) Samples in CRC Patients (*p*-value corrected < 0.05). Functional pathway enrichment analysis shows the enriched metabolic and biosynthetic pathways in T1 compared to S2 samples, based on PICRUSt2 analysis. Pathways are ranked by effect size, with key pathways such as amino acid biosynthesis, and lipopolysaccharide (LPS) biosynthesis significantly enriched in T1. Error bars represent the 95% confidence intervals for differences in pathway proportions.
