# Supplementary figures and images for: Comparative microbiome analysis of paired mucosal and fecal samples in Korean colorectal cancer patients
Source: Front Oncol. 2025 Jun 18;15:1578861. doi: 10.3389/fonc.2025.1578861 (PMC12213350; doi:10.3389/fonc.2025.1578861)

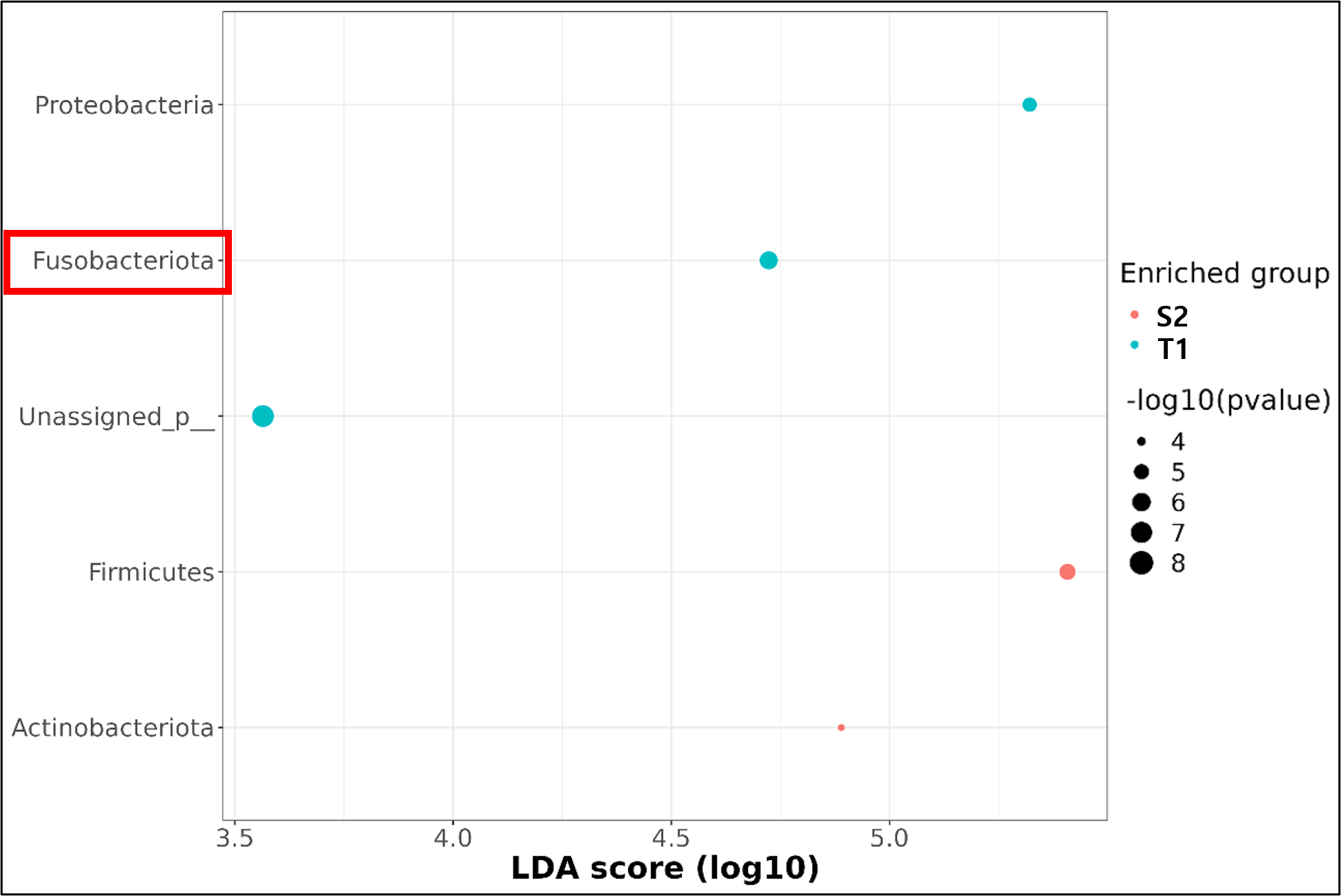

Supplement: Supplementary Figure 1 — Utilizing Linear Discriminant Analysis Effect Size (LEfSe) to Explore Microbial Taxa Differences Between Pre-Surgery and Post-Surgery CRC Patient Samples. (A) Phylum Level Enrichment in tissue samples (T1) Samples. (B) Phylum Level Enrichment in S1 Samples. (C) Venn diagram represented the number of markers for CRC at the Phylum Level between T1 and S1 Samples. [file Image1.tif]

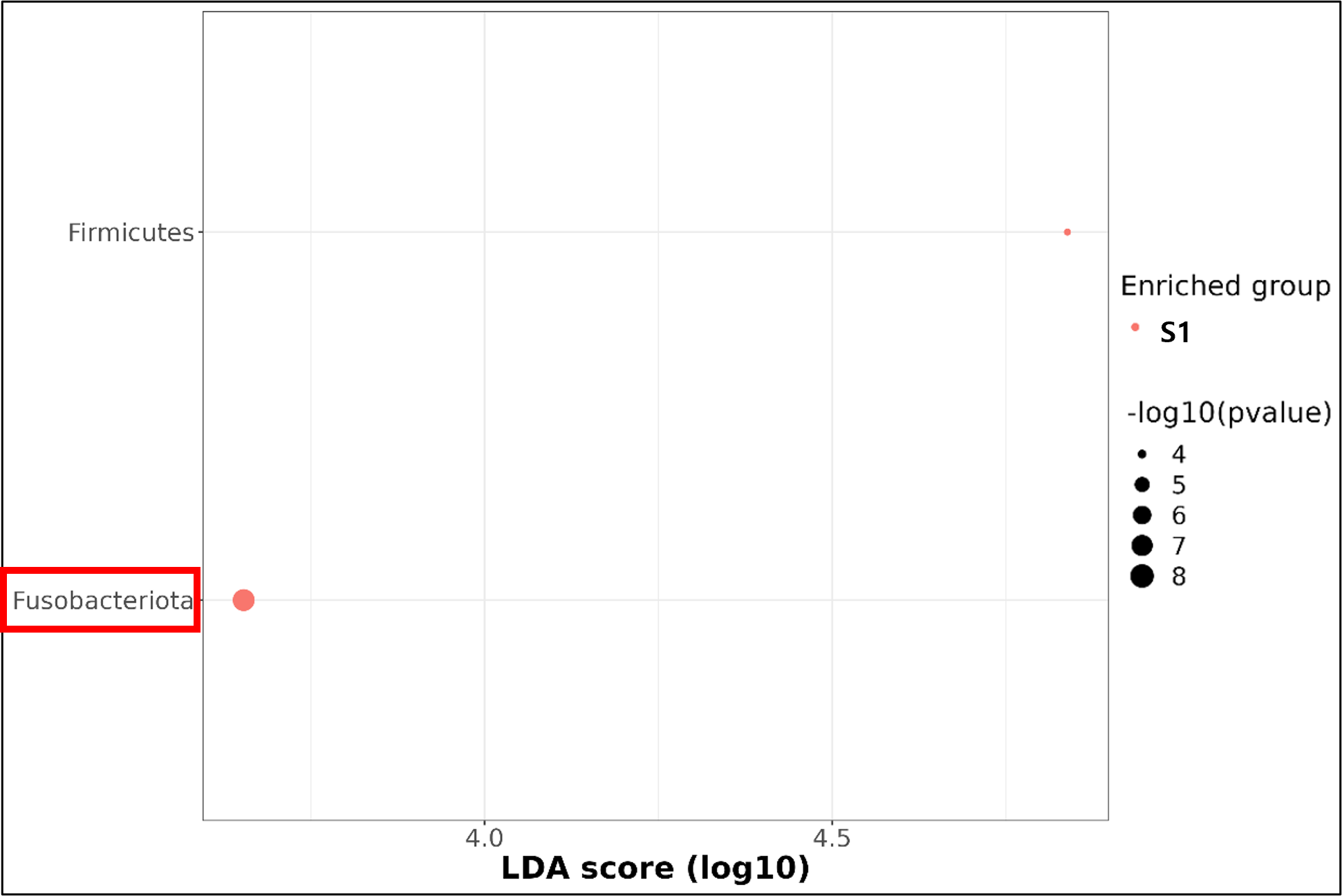

Supplement: Supplementary Figure 2 — Differential Functional Pathway Enrichment Between Mucosal (T1) and Post-Surgery Fecal (S2) Samples in CRC Patients (p-value corrected < 0.05). Functional pathway enrichment analysis shows the enriched metabolic and biosynthetic pathways in T1 compared to S2 samples, based on PICRUSt2 analysis. Pathways are ranked by effect size, with key pathways such as amino acid biosynthesis, and lipopolysaccharide (LPS) biosynthesis significantly enriched in T1. Error bars represent the 95% confidence intervals for differences in pathway proportions. [file Image2.tif]

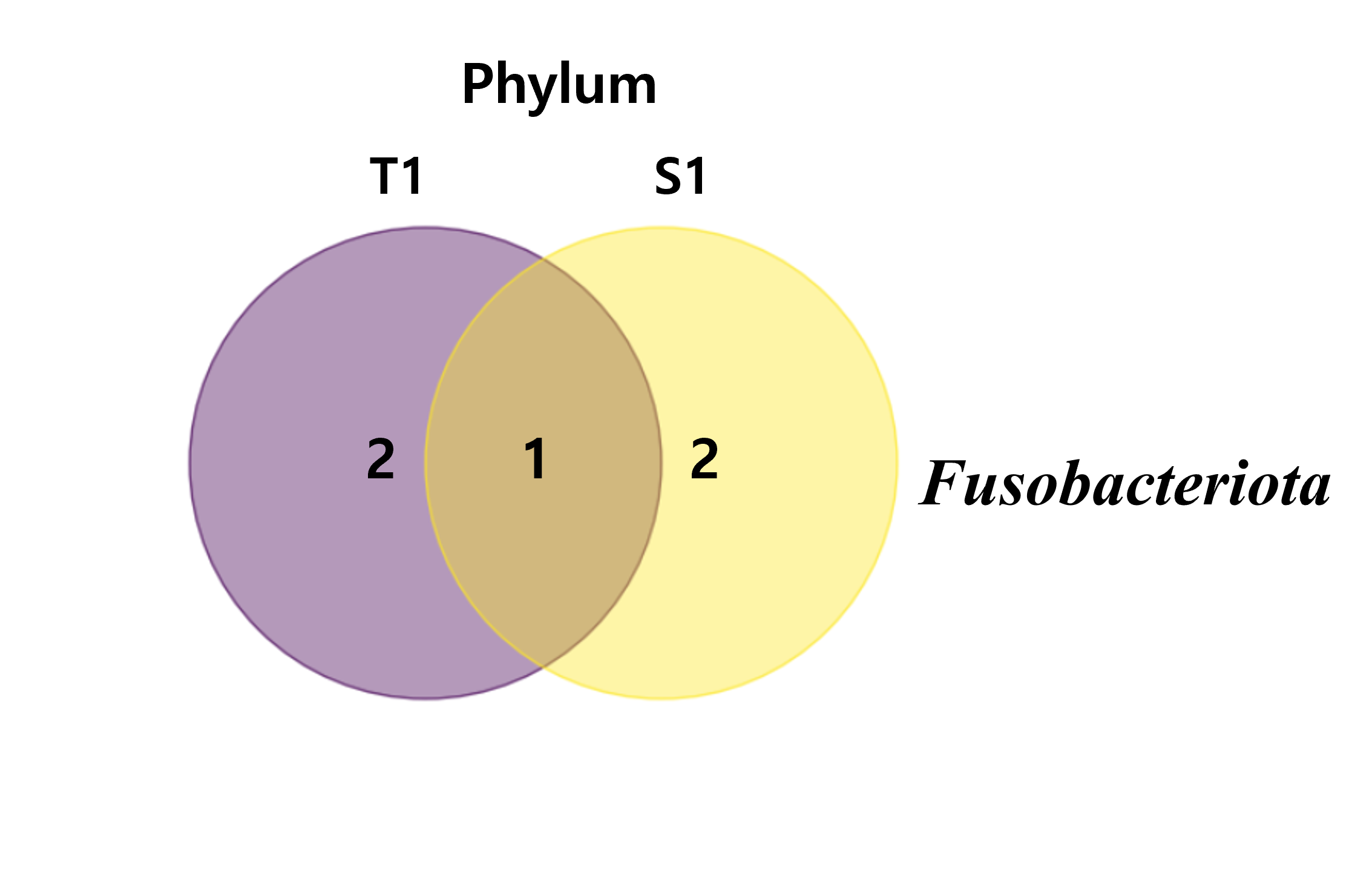

Supplement: Supplementary file 3 [file Image3.tif]

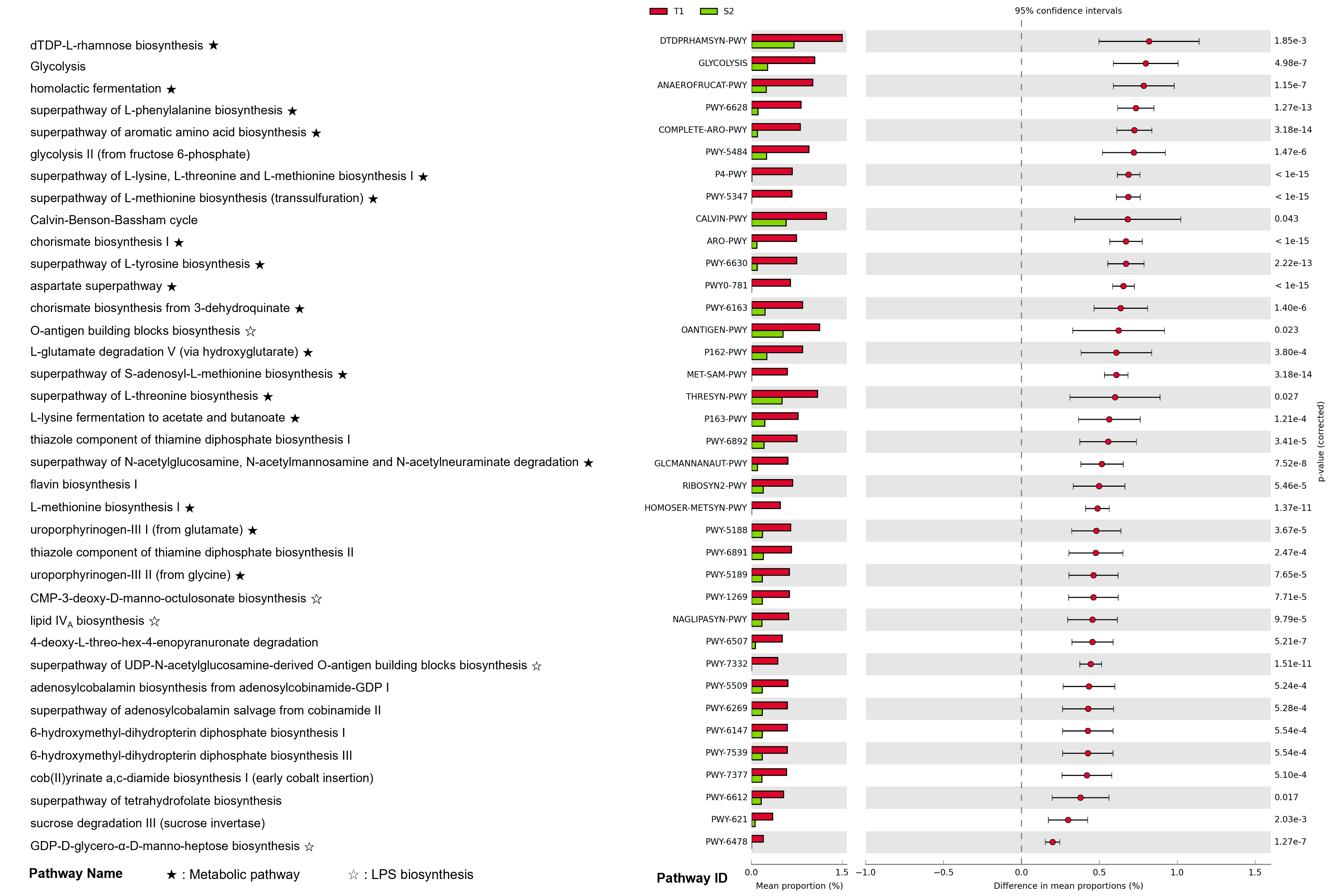

Supplement: Supplementary file 4 [file Image4.tif]
