# Supplementary material for: Comparative microbiome analysis of paired mucosal and fecal samples in Korean colorectal cancer patients
Source: Front Oncol. 2025 Jun 18;15:1578861. doi: 10.3389/fonc.2025.1578861 (PMC12213350; doi:10.3389/fonc.2025.1578861)
Supplement: Supplementary file 5 [file Table1.docx]

Supplementary Table 1. Correlations Between Clinical Variables and Microbial Abundance in Colorectal Cancer Patients. Standard errors and *p*-values were determined through stepwise regression analysis optimized by the Akaike Information Criterion. the *p*-values were adjusted for multiple comparisons using the False Discovery Rate methods. **p*<0.05, ***p*<0.005

SE = Standard errors; HTN = Hypertension; BMI = Body Mass Index; TNM = Tumor Lymphnode Metastasis; HL = Hyperlipidemia; T2DM = Type 2 diabetes mellitus; CEA = Carcinoembryonic antigen; NLR = Neutrophil-to-lymphocyte ratio.

| *Prevotella 9* | Tissue (T1) | | | Pre-surgery (S1) | | |
| --- | --- | --- | --- | --- | --- | --- |
| Clinical variables | BETA | SE | *p*-value | BETA | SE | *p*-value |
| Age | -0.653 | 0.207 | 6.10E-03** | 0.166 | 0.161 | 3.18E-01 |
| Gender (male) | -1.976 | 3.628 | 5.94E-01 | -0.314 | 2.827 | 9.13E-01 |
| HTN | 7.976 | 3.757 | 4.97E-02* | -2.498 | 2.927 | 4.06E-01 |
| Smoking | 3.730 | 3.423 | 2.92E-01 | -2.225 | 2.667 | 4.16E-01 |
| Alcohol | -2.821 | 3.274 | 4.02E-01 | 5.702 | 2.551 | 4.00E-02* |
| BMI | -1.002 | 0.562 | 9.35E-02 | 0.258 | 0.438 | 5.64E-01 |
| Location (right) | -1.343 | 3.277 | 6.87E-01 | -3.220 | 2.553 | 2.25E-01 |
| TNM | -3.162 | 2.302 | 1.89E-01 | 0.722 | 1.794 | 6.39E-01 |
| HL | -5.747 | 3.564 | 1.26E-01 | -1.013 | 2.777 | 7.20E-01 |
| T2DM | -3.366 | 2.825 | 2.51E-01 | 1.930 | 2.201 | 3.93E-01 |
| CEA | 7.034 | 3.535 | 6.39E-02 | -3.977 | 2.754 | 1.68E-01 |
| NLR | -1.482 | 0.852 | 1.01E-01 | -0.775 | 0.664 | 2.60E-01 |
| Probiotics | 0.876 | 3.550 | 8.08E-01 | -1.099 | 2.766 | 6.96E-01 |
