# Supplementary material for: Comparative microbiome analysis of paired mucosal and fecal samples in Korean colorectal cancer patients
Source: Front Oncol. 2025 Jun 18;15:1578861. doi: 10.3389/fonc.2025.1578861 (PMC12213350; doi:10.3389/fonc.2025.1578861)
Supplement: Supplementary file 6 [file Table2.docx]

Supplementary Table 2. Correlations Between Clinical Variables and Microbial Abundance in Colorectal Cancer Patients. Standard errors and p-values were determined through stepwise regression analysis optimized by the Akaike Information Criterion. the p-values were adjusted for multiple comparisons using the False Discovery Rate methods. *p<0.05, **p<0.005

SE = Standard errors; HTN = Hypertension; BMI = Body Mass Index; TNM = Tumor Lymphnode Metastasis; HL = Hyperlipidemia; T2DM = Type 2 diabetes mellitus; CEA = Carcinoembryonic antigen; NLR = Neutrophil-to-lymphocyte ratio.

| *Fusobacterium* | Tissue (T1) | | | Pre-surgery (S1) | | |
| --- | --- | --- | --- | --- | --- | --- |
| Clinical variables | BETA | SE | *p*-value | BETA | SE | *p*-value |
| Age | -0.074 | 0.200 | 7.15E-01 | -0.016 | 0.023 | 5.05E-01 |
| Gender (male) | -5.103 | 3.502 | 1.64E-01 | -0.812 | 0.398 | 5.82E-02 |
| HTN | 1.780 | 3.625 | 6.30E-01 | -0.637 | 0.412 | 1.42E-01 |
| Smoking | 3.331 | 3.304 | 3.28E-01 | 0.738 | 0.376 | 6.71E-02 |
| Alcohol | 2.719 | 3.160 | 4.02E-01 | -0.084 | 0.359 | 8.19E-01 |
| BMI | -0.380 | 0.542 | 4.93E-01 | 0.021 | 0.062 | 7.33E-01 |
| Location (right) | -1.044 | 3.162 | 7.46E-01 | -0.006 | 0.359 | 9.86E-01 |
| TNM | 1.124 | 2.222 | 6.20E-01 | 0.610 | 0.253 | 2.80E-02* |
| HL | -1.213 | 3.439 | 7.29E-01 | 0.322 | 0.391 | 4.22E-01 |
| T2DM | 1.821 | 2.726 | 5.14E-01 | 0.276 | 0.310 | 3.87E-01 |
| CEA | -2.919 | 3.411 | 4.05E-01 | -0.294 | 0.388 | 4.59E-01 |
| NLR | -0.201 | 0.822 | 8.10E-01 | 0.062 | 0.094 | 5.19E-01 |
| Probiotics | 2.012 | 3.427 | 5.65E-01 | -0.455 | 0.389 | 2.60E-01 |
