# Supplementary material for: Comparative microbiome analysis of paired mucosal and fecal samples in Korean colorectal cancer patients
Source: Front Oncol. 2025 Jun 18;15:1578861. doi: 10.3389/fonc.2025.1578861 (PMC12213350; doi:10.3389/fonc.2025.1578861)
Supplement: Supplementary file 7 [file Table3.docx]

Supplementary Table 3. Correlations Between Clinical Variables and Microbial Abundance in Colorectal Cancer Patients. Standard errors and p-values were determined through stepwise regression analysis optimized by the Akaike Information Criterion. the p-values were adjusted for multiple comparisons using the False Discovery Rate methods. *p<0.05, **p<0.005

SE = Standard errors; HTN = Hypertension; BMI = Body Mass Index; TNM = Tumor Lymphnode Metastasis; HL = Hyperlipidemia; T2DM = Type 2 diabetes mellitus; CEA = Carcinoembryonic antigen; NLR = Neutrophil-to-lymphocyte ratio.

| *Parvimonas* | Tissue (T1) | | | Pre-surgery (S1) | | |
| --- | --- | --- | --- | --- | --- | --- |
| Clinical variables | BETA | SE | *p*-value | BETA | SE | *p*-value |
| Age | -0.033 | 0.061 | 5.97E-01 | 0.012 | 0.010 | 2.61E-01 |
| Gender (male) | -0.980 | 1.072 | 3.74E-01 | -0.059 | 0.183 | 7.51E-01 |
| HTN | 0.322 | 1.109 | 7.75E-01 | -0.239 | 0.189 | 2.25E-01 |
| Smoking | -0.624 | 1.011 | 5.46E-01 | -0.107 | 0.172 | 5.45E-01 |
| Alcohol | -0.637 | 0.967 | 5.20E-01 | 0.143 | 0.165 | 3.98E-01 |
| BMI | -0.013 | 0.166 | 9.39E-01 | 0.008 | 0.028 | 7.91E-01 |
| Location (right) | -0.239 | 0.968 | 8.08E-01 | 0.065 | 0.165 | 7.01E-01 |
| TNM | -0.786 | 0.680 | 2.64E-01 | -0.078 | 0.116 | 5.11E-01 |
| HL | -0.978 | 1.052 | 3.67E-01 | 0.238 | 0.180 | 2.03E-01 |
| T2DM | -0.098 | 0.834 | 9.08E-01 | 0.191 | 0.142 | 1.98E-01 |
| CEA | 0.639 | 1.044 | 5.49E-01 | 0.075 | 0.178 | 6.78E-01 |
| NLR | -0.114 | 0.252 | 6.56E-01 | 0.002 | 0.043 | 9.61E-01 |
| Probiotics | -0.491 | 1.049 | 6.46E-01 | -0.022 | 0.179 | 9.02E-01 |
