# Supplementary material for: Comparative microbiome analysis of paired mucosal and fecal samples in Korean colorectal cancer patients
Source: Front Oncol. 2025 Jun 18;15:1578861. doi: 10.3389/fonc.2025.1578861 (PMC12213350; doi:10.3389/fonc.2025.1578861)
Supplement: Supplementary file 8 [file Table4.docx]

Supplementary Table 4. Correlations Between Clinical Variables and Microbial Abundance in Colorectal Cancer Patients. Standard errors and p-values were determined through stepwise regression analysis optimized by the Akaike Information Criterion. the p-values were adjusted for multiple comparisons using the False Discovery Rate methods. *p<0.05

SE = Standard errors; HTN = Hypertension; BMI = Body Mass Index; TNM = Tumor Lymphnode Metastasis; HL = Hyperlipidemia; T2DM = Type 2 diabetes mellitus; CEA = Carcinoembryonic antigen; NLR = Neutrophil-to-lymphocyte ratio.

| *Holdemanella* | Tissue (T1) | | | Pre-surgery (S1) | | |
| --- | --- | --- | --- | --- | --- | --- |
| Clinical variables | BETA | SE | *p*-value | BETA | SE | *p*-value |
| Age | -0.041 | 0.027 | 1.56E-01 | -0.030 | 0.034 | 3.80E-01 |
| Gender (male) | 0.944 | 0.477 | 6.53E-02 | 0.319 | 0.588 | 5.95E-01 |
| HTN | 0.145 | 0.494 | 7.73E-01 | 0.988 | 0.609 | 1.24E-01 |
| Smoking | 0.194 | 0.449 | 6.73E-01 | -5.12E-05 | 0.555 | 9.99E-01 |
| Alcohol | -0.077 | 0.430 | 8.59E-01 | 0.355 | 0.531 | 5.13E-01 |
| BMI | 0.046 | 0.074 | 5.39E-01 | -0.020 | 0.091 | 8.28E-01 |
| Location (right) | 0.460 | 0.431 | 3.01E-01 | 0.166 | 0.531 | 7.59E-01 |
| TNM | -0.035 | 0.302 | 9.08E-01 | -0.299 | 0.373 | 4.35E-01 |
| HL | -1.138 | 0.468 | 2.72E-02* | -0.721 | 0.578 | 2.30E-01 |
| T2DM | -0.460 | 0.371 | 2.33E-01 | -0.182 | 0.458 | 6.96E-01 |
| CEA | 0.237 | 0.464 | 6.17E-01 | 1.172 | 0.573 | 5.76E-02 |
| NLR | -0.008 | 0.112 | 9.47E-01 | 0.004 | 0.138 | 9.79E-01 |
| Probiotics | 0.905 | 0.466 | 7.03E-02 | 0.406 | 0.576 | 4.90E-01 |
